# *SLC22A3* polymorphisms do not modify pancreatic cancer risk, but may influence overall patient survival

**DOI:** 10.1038/srep43812

**Published:** 2017-03-08

**Authors:** Beatrice Mohelnikova-Duchonova, Ondrej Strouhal, David J. Hughes, Ivana Holcatova, Martin Oliverius, Zdenek Kala, Daniele Campa, Cosmeri Rizzato, Federico Canzian, Raffaele Pezzilli, Renata Talar-Wojnarowska, Ewa Malecka-Panas, Cosimo Sperti, Carlo Federico Zambon, Sergio Pedrazzoli, Paola Fogar, Anna Caterina Milanetto, Gabriele Capurso, Gianfranco Delle Fave, Roberto Valente, Maria Gazouli, Giuseppe Malleo, Rita Teresa Lawlor, Oliver Strobel, Thilo Hackert, Nathalia Giese, Pavel Vodicka, Ludmila Vodickova, Stefano Landi, Francesca Tavano, Domenica Gioffreda, Ada Piepoli, Valerio Pazienza, Andrea Mambrini, Mariangela Pedata, Maurizio Cantore, Franco Bambi, Stefano Ermini, Niccola Funel, Radmila Lemstrova, Pavel Soucek

**Affiliations:** 1 Department of Toxicogenomics, National Institute of Public Health, Prague, Czech Republic; 2 Department of Oncology, Palacky University Medical School and Teaching Hospital, Olomouc, Czech Republic; 3 Department of Physiology & Centre for Systems Medicine, Royal College of Surgeons in Ireland, Dublin 2, Ireland; 4 Institute of Hygiene and Epidemiology, First Faculty of Medicine, Charles University in Prague, Prague, Czech Republic; 5 Department of Transplantation Surgery, Institute of Clinical and Experimental Medicine, Prague, Czech Republic; 6 Department of Surgery, The University Hospital and Faculty of Medicine, Brno Bohunice, Czech Republic; 7 Genomic Epidemiology Group, German Cancer Research Center (DKFZ), Heidelberg, Germany; 8 Department of Biology, University of Pisa, Pisa, Italy; 9 Department of Translational Research and New Technologies in Medicine and Surgery, University of Pisa, Pisa, Italy; 10 Department of Digestive Diseases, Sant’Orsola-Malpighi Hospital, Bologna, Italy; 11 Department of Digestive Tract Diseases, Medical University of Lodz, Lodz, Poland; 12 Department of Surgery, Oncology and Gastroenterology -DiSCOG, University of Padova, Italy; 13 Department of Medicine - DIMED, University of Padova, Italy; 14 Clinica Chirurgica 4, University of Padova, Italy; 15 Department of Laboratory Medicine, University-Hospital of Padova, Italy; 16 Digestive and Liver Disease Unit, S. Andrea Hospital, ‘Sapienza’ University of Rome, Rome, Italy; 17 Department of Basic Medical Sciences, Laboratory of Biology, School of Medicine, University of Athens, Athens, Greece; 18 Department of Surgery and Oncology, University and Hospital Trust of Verona, Verona, Italy; 19 ARC-NET Applied research on Cancer Centre, University and Hospital Trust of Verona, Verona, Italy; 20 Department of General, Visceral and Transplantation Surgery, Heidelberg University Hospital, Heidelberg, Germany; 21 Department of Molecular Biology of Cancer, Institute of Experimental Medicine, Academy of Science of Czech Republic, Prague, Czech Republic and First Faculty of Medicine, Charles University in Prague, Czech Republic; 22 Biomedical Centre, Faculty of Medicine in Pilsen, Charles University in Prague, Pilsen, Czech Republic; 23 Division of Gastroenterology and Research Laboratory, IRCCS Scientific Institute and Regional General Hospital “Casa Sollievo della Sofferenza”, San Giovanni Rotondo, Italy; 24 Department of Oncology, Azienda USL 1 Massa Carrara, Massa Carrara, Italy; 25 Blood Transfusion Service, Children’s Hospital Meyer, Azienda Ospedaliero Universitaria, Florence, Italy

## Abstract

Expression of the solute carrier (SLC) transporter SLC22A3 gene is associated with overall survival of pancreatic cancer patients. This study tested whether genetic variability in SLC22A3 associates with pancreatic cancer risk and prognosis. Twenty four single nucleotide polymorphisms (SNPs) tagging the SLC22A3 gene sequence and regulatory elements were selected for analysis. Of these, 22 were successfully evaluated in the discovery phase while six significant or suggestive variants entered the validation phase, comprising a total study number of 1,518 cases and 3,908 controls. In the discovery phase, rs2504938, rs9364554, and rs2457571 SNPs were significantly associated with pancreatic cancer risk. Moreover, rs7758229 associated with the presence of distant metastases, while rs512077 and rs2504956 correlated with overall survival of patients. Although replicated, the association for rs9364554 did not pass multiple testing corrections in the validation phase. Contrary to the discovery stage, rs2504938 associated with survival in the validation cohort, which was more pronounced in stage IV patients. In conclusion, common variation in the SLC22A3 gene is unlikely to significantly contribute to pancreatic cancer risk. The rs2504938 SNP in SLC22A3 significantly associates with an unfavorable prognosis of pancreatic cancer patients. Further investigation of this SNP effect on the molecular and clinical phenotype is warranted.

Pancreatic ductal adenocarcinoma (PDAC, OMIM: 260350) has an extremely poor prognosis^1^ mostly due to the late diagnosis of disease, when all treatment options are limited. Thus, it is imperative to improve prevention and early detection efforts, such as locating genetic markers of PDAC risk that could inform early detection of the disease.

There are several established epidemiological risk factors for PDAC, e.g., smoking, obesity, personal history of chronic pancreatitis or diabetes, and family history of cancers^2^. A small fraction of PDACs are caused by high-risk predisposing mutations in DNA repair and damage sensing genes, e.g., *BRCA1, BRCA2, PALB2, ATM, CDKN2A, APC, MLH1, MSH2, MSH6, PMS2, PRSS1*, and *STK11*^3^. Several genome-wide association studies (GWAS) have identified several low-penetrance loci associating with PDAC risk^4^,^5^,^6^,^7^. These authors estimated that the current loci identified in European populations account for approximately only 5% of the inherited pancreatic cancer risk, indicating that a large portion of familial risk alleles remain to be revealed.

The role of the SLC22A subfamily of solute carrier (SLC) transporters in PDAC progression is, at present, not well understood. SLC22A1, SLC22A2, and SLC22A3 mediate the transport of a variety of structurally diverse cations comprising both endogenous and exogenous compounds, e.g., neurotransmitters such as catecholamines and xenobiotics (including drugs), respectively^8^,^9^.

Our recent study revealed a highly significant upregulation of *SLC22A3* transcripts in PDAC tumors compared with non-neoplastic tissues^10^. Moreover, a high level of *SLC22A3* mRNA in tumors strongly predicted a longer overall survival (*P* = 0.004) in chemotherapy-treated patients. Association studies also suggest that genetic variability in *SLC22A3* is likely to be associated with the risk of different cancer types. A colorectal cancer GWAS reported that rs7758229 in the *SLC22A3* gene (Gene ID: 6581) was significantly associated with distal colon cancer risk in Asians^11^. Interestingly, SNP rs9364554 in intron 5 of SLC22A3 was previously shown to associate with prostate cancer in Caucasian populations^12^ suggesting a pleiotropic effect of the SNP.

This study tested the hypothesis that genetic variation in the *SLC22A3* gene contributes to pancreatic cancer risk and disease survival. The tagging approach of the whole *SLC22A3* gene region, including regulatory elements, was used in a two-stage genetic association study of Europeans.

## Results and Discussion

### Associations of SLC22A3 SNPs with pancreatic cancer risk

In this study, an association analysis of SNPs tagging the *SLC22A3* gene with PDAC risk was performed in a two-stage design comprising in total 1,254 cases and 3,391 controls of European descent (Table 1).

We genotyped 208 PDAC cases and 381 controls from the Czech Republic in the discovery phase^13^,^14^. Three SNPs (rs2504938, rs9364554, and rs2457571) were significantly associated with PDAC risk in at least one of the genetic models tested (Table 2). These three SNPs were further analyzed in the validation phase comprising 1,046 and 3,010 controls from the PANDoRA (PANcreatic Disease ReseArch), European case-control study of PDAC^15^. A significant association for rs9364554 (OR = 1.19, 95% CI = 1.02–1.40, p = 0.030) was observed (Table 3), which did not pass the FDR test (q = 0.008) for correction of multiple comparisons. Moreover, combined analysis of both sets rendered all associations as non-significant (Supplementary Table S1). Thus, the results suggest that genetic variability in *SLC22A3* probably does not significantly contribute to PDAC risk in the European population.

The trends observed by univariate analyses in the discovery stage did not change in the multivariate analyses adjusted to age, sex, body mass index (BMI), smoking status, and alcohol consumption with the exception of rs4708867 where carriage of the rare G allele was associated with an increased PDAC risk (Supplementary Table S2). Due to the lack of lifestyle data in the validation phase this association could not be replicated.

The present study attempted, for the first time, to find a link between the genetic variability in the *SLC22A3* gene and PDAC risk. The recently reported GWAS on PDAC risk did not find any association with *SLC22A3* tagging variants at genome wide significance^6^,^7^. Thus, despite previous reports on association of rs7758229 SNP with colorectal cancer risk^11^ and rs9364554 with prostate cancer risk^12^,^16^ the present study confirms that common genetic variability in *SLC22A3* most probably does not modify PDAC risk. Cancer-specific effects, largely unknown gene-environmental interactions, and inter-population (even between Europeans) heterogeneity may underlie the observed differences.

### Associations of SLC22A3 SNPs with pancreatic cancer survival

The second goal of this study was to assess whether genetic variability in *SLC22A3* associates with major clinical characteristics of PDAC considering our previously reported association of intratumoral *SLC22A3* gene expression with overall survival of PDAC patients^10^. In the discovery phase, the number of carriers of the T allele or heterozygous genotype in rs7758229 was significantly higher in patients with metastatic disease than in those without distant metastases (OR = 3.63, 95% CI = 1.46–9.06, p = 0.006 for heterozygotes compared with the GG genotype carriers and OR = 2.76, 95% CI = 1.20–6.32, p = 0.016 for T allele carriers compared with the GG genotype carriers, Table 4). Development of metastases is a major sign of cancer spread due to aggressive behavior of the tumor and predicts poor prognosis. Therefore, this SNP was added to the list of SNPs for validation.

**Figure 1:**
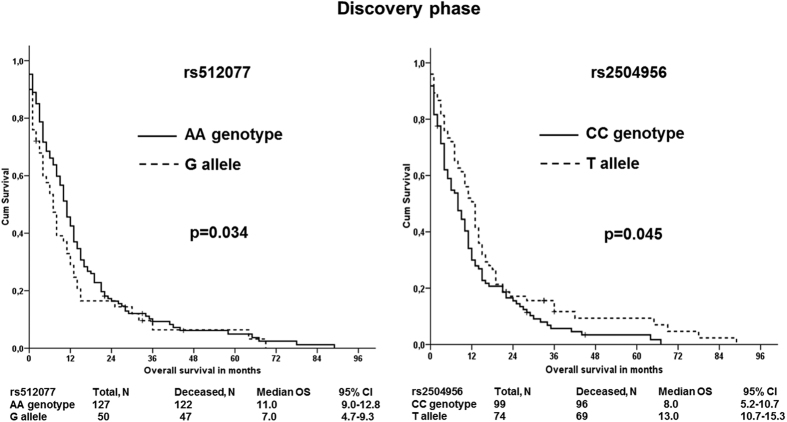
Associations of *SLC22A3* rs512077 and rs2504956 SNPs with overall survival of PDAC patients in the discovery phase Kaplan–Meier survival curves for patients with wild type (solid line) *vs.* patients with rare allele (dashed line) are displayed. The difference in the mean survival between the compared groups of patients was significant (p = 0.034 and p = 0.045 for rs512077 and rs2504956 SNPs, respectively). Hazard ratios, 95% confidence intervals, and p-values calculated by the stage-adjusted Cox regression are presented in Supplementary Table S3.

Additionally, patients carrying the AA genotype in rs512077 or T allele in rs2504956 had significantly better overall survival than the other patients (Fig. 1), suggesting that these SNPs might serve as markers of PDAC patient prognosis. None of the other analyzed SNPs in the discovery set were significantly associated with disease outcomes.

**Figure 2:**
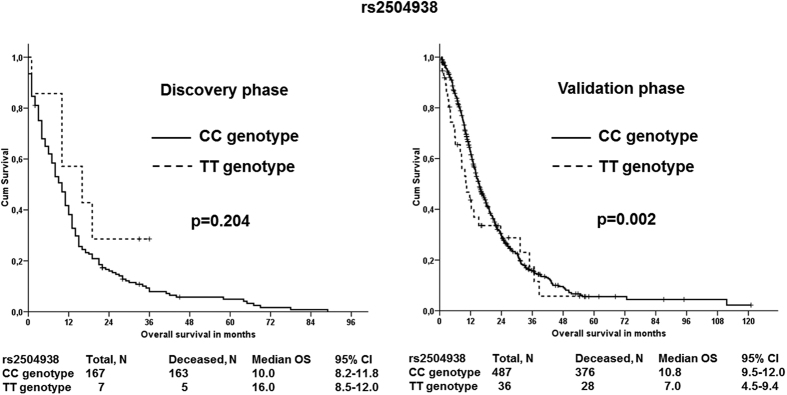
Associations of *SLC22A3* rs2504938 SNP with overall survival of PDAC patients in both phases Kaplan–Meier survival curves for patients with CC genotype (solid line) *vs.* patients with TT genotype (dashed line) are displayed. The difference in the mean survival between the compared groups of patients was significant (p = 0.002) in the validation phase. Hazard ratios, 95% confidence intervals, and p-values calculated by the stage-adjusted Cox regression are presented in Supplementary Table S3.

**Figure 3:**
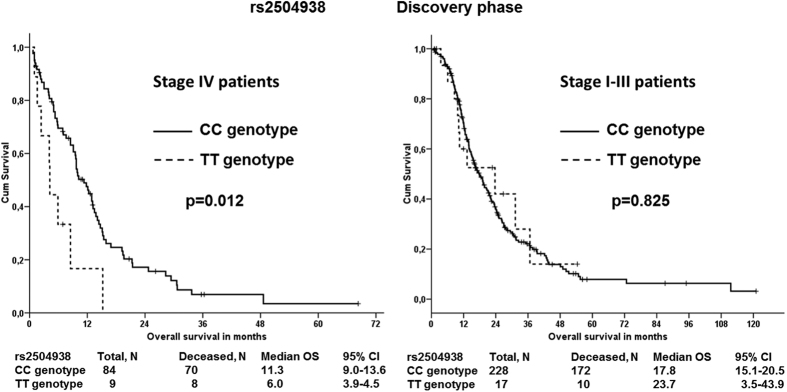
Stage-stratified associations of *SLC22A3* rs2504938 SNP with overall survival of PDAC patients in the discovery phase Kaplan–Meier survival curves for patients with CC genotype (solid line) *vs.* patients with TT genotype (dashed line) are displayed for patients with stage IV (left) or stage I–III (right) disease. The difference in the mean survival between the compared groups of patients with stage IV disease was significant (p = 0.012).

None of these associations with either metastasis or survival were replicated in the validation phase (all p-values > 0.05). However, patients carrying the TT genotype in rs2504938 had highly significantly worse overall survival than patients with the CC genotype in the validation set (p = 0.002, Fig. 2) which notably retains significance after FDR correction for multiple testing in the validation phase (q = 0.008). However, the combined analysis of discovery and validation sets was not significant (p = 0.073, Supplementary Figure S1). Stage-adjusted analysis of all three SNPs (rs512077, rs2504956, and rs2504938) in both sets separately and combined also showed no significant associations (Supplementary Table S3). When analyzing patients stratified by stage, we observed that patients with stage IV disease carrying the TT genotype in rs2504938 had significantly worse OS than CC genotype carriers (p = 0.012, Fig. 3). In patients with less advanced disease (stages I–III) no such association was found (Fig. 3).

LD analysis in cases from validation phase suggested that rs2504956 and rs512077 are in high LD (r^2^ = 0.95), rs2504938 and rs2504956 in strong LD (r^2^ = 0.81) and rs2504938 and rs512077 in weak LD (r^2^ = 0.42). This analysis strengthens the observed genetic link of *SLC22A3* polymorphisms (rs2504956 in the discovery set and rs2504938 in the validation set) with the OS of PDAC patients. A more refined study of these two loci and surrounding sequences may shed more light into the prognostic importance of *SLC22A3* variability in PDAC.

A potential functional effect of the rs2504938 and rs2504956 SNPs was tested by two ways. First, we analyzed the *in silico* prediction by HaploReg v3 indicating that rs2504938 may alter motifs for DNA binding proteins and transcription factors Hmx_1 (H6 Family Homeobox 1 DNA binding protein, OMIM: 142992) and NF-kappaB_known3 (Nuclear Factor Kappa B, OMIM: 164011). Additionally, rs2504956 may alter motifs E2A_2 and E2A_5 (Transcription Factor 3, OMIM: 147141), Hic1_1 (Hypermethylated in Cancer 1, OMIM: 603825), and ZBTB7A_known1 (Zinc Finger- and BTB Domain-containing Protein 7 A, OMIM: 605878). Second, we analyzed whether rs2504938 allele distribution correlates with gene expression of *SLC22A3* in tumor (n = 17) and paired adjacent non-malignant tissues (n = 15) of the subgroup of patients assessed by our previous study^10^. Although there were modest tissue sample numbers available, the comparison suggests no significant correlation of rs2504938 or rs2504956 with *SLC22A3* expression in PDAC tissues (p > 0.05). Thus, any potential influence of the rs2504938 SNP on PDAC survival would not appear to act via the gene expression level. Alternatively, a currently unknown link with other, potentially functional, genetic variation may explain the observed association with survival of PDAC patients.

Moreover, in the light of recent findings demonstrating that neurotransmitters help stimulate prostate tumor growth and metastasis^17^ and accelerate pancreatic cancer cell growth and invasion^18^, it would be worthwhile examining whether SLC22A3 might be involved in cancer tumorigenesis through the clearance of these active compounds.

As our previous study showed an association of *SLC22A3* gene expression with overall survival only in patients treated with nucleoside analogs^10^, it would be very interesting to perform a survival analysis stratified by therapy. However, PANDoRA does not yet have sufficiently relevant data for such an analysis at present. Together with this, variations in age in both sets and BMI distribution in the discovery set are limitations of this study. Although, there were no considerable differences between crude and adjusted analyses of both sets, we cannot exclude a potential for some false negative findings. Future meta-analysis of results of this and subsequent independent studies will help to further evaluate these reported associations.

In conclusion, the present study suggests that common genetic variability in the *SLC22A3* gene is not significantly associated with risk of PDAC. The rs2504938 SNP was associated with overall survival in the large PANDoRA study when evaluated in univariate manner and especially in stage IV patients, although the biological basis of this correlation remains to be elucidated.

## Subjects and Methods

### Study populations

We used a two-step strategy with a discovery phase consisting of biological samples from 245 PDAC patients (cases) and 442 controls of Czech Caucasian origin collected in the Czech Republic between 2004 and 2010. Patients were eligible for the study, when they fulfilled at least one of the following criteria: (a) patient had histology- or cytology-confirmed pancreatic adenocarcinoma or (b) at least three of clinical signs of pancreatic cancer (ERCP, EUS with FNAB, mass on CT or MRI, weight loss, anorexia/cachexia, obstructive jaundice). Clinical and pathological data on the cases (date of diagnosis, stage, grade, and histologic diagnosis where available) were collected from their medical records. The controls were included into the study under the condition that the difference in their age was not larger than 5 years from cases recruited in the same period. Basic epidemiological data on all participants (personal history, smoking and drinking history, physical activity, occupational and nutritional information) were collected (for case and control recruitment criteria see refs 13,14). The validation phase consisted of 1,273 cases and 3,466 controls enrolled into the PANcreatic Disease ReseArch (PANDoRA) consortium from three other European countries (Germany, Italy, and Poland). For all cases and controls a DNA sample from blood and/or pancreatic tissue was available, as well as a minimal set of covariates (such as age at diagnosis, sex, disease stage, age of death or at last follow-up for majority of cases). Different region-specific subpopulations of unmatched controls have been selected among the general population, blood donors and among hospitalized subjects with different diagnosis excluding cancer (described in detail in ref. 15).

There are no relevant data concerning chemotherapy treatments and responses so far. Relevant baseline characteristics of the studied populations are shown in Table 1.

Informed consent was obtained from all participating subjects for these studies in accord with the Declaration of Helsinki. All samples were coded to protect patient anonymity. The study was approved by the Ethical Committee of the University of Heidelberg (reference number S-565/2015). All methods were performed in accordance with guidelines and regulations set by the above Ethical Committee.

### Selection of polymorphisms

The *SLC22A3* gene region together with 10 kb sequences flanking the 5′ and 3′ ends (chr6: chr6:160680000…160806000, NCBI assembly 36) was analyzed by HaploView v4.2 program using a pairwise tagging approach with r^2^ > 0.8^19^. SNPs with minor allele frequency (MAF) > 0.01 in HapMap CEU sample (International HapMap Project, version 28; http://www.hapmap.org) and at least 75% genotype data were identified. Together 24 SNPs tagging 139 alleles in the analyzed region were selected for analysis in the discovery phase.

The chromosomal locations and minor allele frequencies of the tested SNP variants are listed in Supplementary Table S4.

### Genotyping

DNA from the cases and controls in the discovery sample set was isolated from peripheral lymphocytes using a BioSprint 15 DNA Blood kit (Qiagen, Valencia, CA) by KingFisher mL automated system (Thermo Electron Corporation, Vantaa, Finland) according to the manufacturer’s protocol. DNA from participants in the PANDoRA cohort was isolated from whole blood using the Qiagen-mini kit or the AllPrep Isolation kit (both Qiagen) using provider’s protocol. DNA was quantified by Quant-iT PicoGreen DNA Assay Kit (Invitrogen).

In the discovery phase, 24 SNPs were analyzed in DNA from 245 PDAC cases and 442 controls of Czech origin using *KASPar* technology (LGC Genomics, Hoddesdon, UK). Validation failed for rs12212246 due to unspecific amplification and therefore this SNP was not further analyzed.

During the validation phase, six candidate SNPs (i.e., those that showed an association in the discovery phase with either the risk or clinical outcomes of PDAC in the Czech cohort) were genotyped in the PANDoRA sample set consisting of 1,273 cases and 3,466 controls of European origin. Genotyping was performed at National Institute of Public Health, Prague, Czech Republic by allelic discrimination using TaqMan technology (Life Technologies Corp., Foster City, CA) in a ViiA7 real-time instrument with a 384-well block (Life Technologies). SNP assay reaction conditions are summarized in Supplementary Table S5.

Quality control was performed by determination of duplicate samples for approximately 10% of the samples in both phases. The genotyping concordance between duplicate samples exceeded 99%. All samples with less than 75% successful genotypes for all SNPs were discarded from further analysis. In total, the generated genotypes for 208 cases and 381 controls in the discovery phase and 1,046 cases and 3,010 controls in the validation phase were then analyzed. Together 23 SNPs were successfully genotyped.

### Statistical analysis

Hardy Weinberg Equilibrium (HWE) was first examined in control subjects for each SNP. Genotype distribution of the studied SNPs did not deviate from HWE (p > 0.05) with the exception of rs3004079. The rs3004079 variant was therefore excluded from further analyses. Unconditional logistic regression was then used to assess the association of the 22 remaining SNPs with PDAC risk in the discovery phase. Co-dominant, dominant, and recessive genetic models were evaluated. Crude and adjusted for age (continuous), sex, and country of origin odds ratios (OR), 95% confidence intervals (CI), and p-values were calculated for each SNP. Age-, sex-, body mass index-, smoking status-, and alcohol consumption-adjusted analyses were performed by logistic regression in the discovery phase.

Associations of SNPs with prognostic clinical data (tumor size, presence of lymph node and distant metastases) were evaluated by the Cochran’s and Mantel-Hanszel statistics. Overall survival (OS) was defined as time elapsed from diagnosis to patient death, or to the last date at which the patient was known to be alive. Patients lost to follow up were excluded from analyses. Survival functions were plotted by the Kaplan-Meier method and statistical significance was evaluated by the Log-rank test. Stage-adjusted hazard ratios were then calculated by Cox regression. A p-value of less than 0.05 was considered statistically significant. Analyses were conducted by the statistical program SPSS v15.0 (SPSS, Chicago, IL).

The six SNPs genotyped in the validation phase were evaluated using the same statistical methods as for the discovery phase.

In multiple testing adjustments, as Bonferroni’s correction was considered too stringent because of linkage disequilibrium (LD) among the SNPs we tested, the Benjamini-Hochberg false discovery rate (FDR) test^20^ was used for the evaluation of results in the validation phase.

The functional relevance of the SNP showing significant association (rs2504938) was analyzed *in silico* by HaploReg v2 and v3^21^. Information about the observed association of this SNP with clinical phenotype of PDAC was submitted to NCBI (The National Center for Biotechnology Information) ClinVar database (http://www.ncbi.nlm.nih.gov/clinvar).

## Additional Information

**How to cite this article:** Mohelnikova-Duchonova, B. *et al. SLC22A3* polymorphisms do not modify pancreatic cancer risk, but may influence overall patient survival. *Sci. Rep.* **7**, 43812; doi: 10.1038/srep43812 (2017).

**Publisher's note:** Springer Nature remains neutral with regard to jurisdictional claims in published maps and institutional affiliations.

## Supplementary Material

Supplementary Dataset 1

## Figures and Tables

**Table 1:** The baseline characteristics of studied groups of individuals with complete genotypes.

Czech sample set	Controls		Cases		p-value^†^
	N	%	N	%	
Age (mean ± S.D.)^*^	51.4 ± 14.5		62.8 ± 10.2		< 0.001
Sex^*^					0.860
Males	229	60.7	128	61.5	
Females	148	39.3	80	38.5	
Body mass index					0.001
(mean ± S.D.)^*^	30.0 ± 4.2	25.4 ± 4.9			
Missing data	89	62			
Smoking status					0.257
Neversmokers	130	45.0	56	38.9	
Smokers including					
former smokers	159	55.0	88	61.1	
Missing data	88	—	64	—	
Alcohol consumption					0.466
Teetotallers	118	40.5	53	39.2	
Drinkers including					
former drinkers	173	59.5	92	60.8	
Missing data	86	—	63	—	
Clinical stage					
Stage I	NA	7	7.4		
Stage IIA	NA	12	12.8		
Stage IIB	NA	15	16.0		
Stage III	NA	13	13.8		
Stage IV	NA	47	50.0		
Missing data	NA	114	—		
Vital status					
Dead	NA	172	95.5		
Alive	NA	8	4.5		
Missing data	NA	28	—		
Pandora sample set					
Age (mean ± S.D.)^#^	60.5 ± 12.2		64.1 ± 11.1		< 0.001
Sex^#^					0.334
Males	1702	56.6	590	56.6	
Females	1303	43.4	453	43.4	
Country and region					
Germany	1032	34.3	94	9.0	
Heidelberg	0		94	—	
Mannheim	1032		0	—	
Italy	1651	53.2	862	80.7	
Bologna	0		70	—	
Carrara	0		209	—	
Florence	435		0	—	
Padua	608		188	—	
Pisa	0		74	—	
Roma	89		109	—	
San Giovanni Rotondo	519		106	—	
Verona	0		106	—	
Poland, Lodz	167	5.5	56	5.3	
Czech Republic, Prague	160	7.0	34	5.0	
Clinical stage					
Stage I	NA		26	4.5	
Stage IIA	NA		64	11.0	
Stage IIB	NA		207	35.7	
Stage III	NA		104	17.9	
Stage IV	NA		179	30.9	
Missing data	NA		466	—	
Vital status					
Dead	NA		611	25.5	
Alive	NA		209	74.5	
Missing data	NA		226	—	

In total, 208 cases and 381 controls in the discovery phase and 1,046 cases and 3,010 controls in the validation phase were analyzed after quality control evaluation.

^*^Information about four controls is missing.

^#^Information about five controls and three cases is missing.

^†^Significance of differences between cases and controls was evaluated by the Mann-Whitney test (sex, smoking status, and alcohol consumption) and by the Fisher’s Exact test (age and body mass index).

NA = not applicable.

**Table 2:** Results of crude analyses of associations of *SLC22A3* SNPs with pancreatic cancer risk in the discovery phase.

SNP	Controls		Cases		OR	95% CI	p
genotype	N	%	N	%			
rs316174 (A>G)							
AA	121	31.9	64	31.2	reference		
AG	182	48.0	107	52.2	1.11	0.76–1.64	0.591
GG	76	20.1	34	16.6	0.85	0.51–1.41	0.516
G allele	258	—	141	—	1.03	0.71–1.49	0.861
A allele^*^	303	—	171	—	0.79	0.51–1.23	0.307
rs2504956 (C>T)							
CC	240	63.2	115	55.8	reference		
CT	122	32.1	81	39.3	1.39	0.97–2.00	0.074
TT	18	4.7	10	4.9	1.16	0.52–2.56	0.719
T allele	140	—	91	—	1.35	0.96–1.92	0.083
C allele^*^	362	—	196	—	1.03	0.47–2.27	0.949
rs572149 (A>G)							
AA	153	41.6	149	42.0	reference		
AG	173	47.0	54	51.2	1.08	0.75–1.54	0.675
GG	42	11.4	5	6.8	0.59	0.31–1.15	0.121
G allele	214	—	59	—	0.98	0.69–1.39	0.930
A allele^*^	326	—	203	—	0.57	0.30–1.08	0.080
rs3120137 (G>A)							
GG	289	75.9	160	77.3	reference		
GA	87	22.8	42	20.3	0.87	0.57–1.32	0.519
AA	5	1.3	5	2.4	1.82	0.52–6.25	0.356
A allele	92	—	47	—	0.93	0.62–1.32	0.694
G allele^*^	376	—	202	—	1.85	0.53–6.67	0.330
rs512077 (A>G)							
AA	255	67.5	149	72.0	reference		
AG	113	29.9	53	25.6	0.80	0.55–1.18	0.262
GG	10	2.6	5	2.4	0.85	0.29–2.56	0.780
G allele	123	—	58	—	0.81	0.56–1.18	0.258
A allele^*^	368	—	202	—	0.91	0.31–2.70	0.866
rs675162 (A>G)							
AA	270	73.2	163	79.9	reference		
AG	88	23.8	40	19.6	0.75	0.50–1.15	0.187
GG	11	3.0	1	0.5	0.15	0.02–1.18	0.071
G allele	99	—	41	—	0.86	0.45–1.04	0.073
A allele^*^	358	—	203	—	0.16	0.02–1.25	0.081
rs394487 (C>T)							
CC	178	47.1	101	48.6	reference		
CT	170	45.0	96	46.2	0.99	0.70–1.41	0.979
TT	30	7.9	11	5.3	0.65	0.31–1.35	0.243
T allele	200	—	107	—	0.94	0.67–1.32	0.734
C allele^*^	348	—	197	—	0.65	0.32–1.32	0.232
rs10455871(A>T)							
AA	369	97.6	199	96.1	reference		
AT	9	2.4	8	3.9	1.64	0.63–4.35	0.312
TT	0	0	0	0	NA	NA	NA
T allele	9	—	8	—	1.64	0.63–4.35	0.312
A allele^*^	378	—	207	—	NA	NA	NA
rs884742 (C>A)							
CC	287	76.3	172	82.7	reference		
CA	82	21.8	35	16.8	0.71	0.46–1.10	0.129
AA	7	1.9	1	0.5	0.24	0.03–1.96	0.182
A allele	89	—	36	—	0.70	0.44–1.04	0.074
C allele*	369	—	207	—	0.25	0.03–2.08	0.202
rs420038 (C>T)							
CC	173	47.0	99	48.8	reference		
CT	165	44.8	91	44.8	0.96	0.68–1.37	0.839
TT	30	8.2	13	6.4	0.76	0.38–1.52	0.434
C allele	195	—	104	—	0.93	0.66–1.32	0.687
T allele^*^	338	—	190	—	0.77	0.39–1.52	0.450
rs1567441 (T>C)							
TT	201	53.2	113	54.6	reference		
TC	159	42.1	83	40.1	0.93	0.65–1.32	0.679
CC	18	4.8	11	5.3	1.09	0.50–2.38	0.835
C allele	177	—	94	—	0.94	0.67–1.33	0.743
T allele^*^	360	—	196	—	1.12	0.52–2.44	0.769
**rs2504938** (C>T)							
CC	234	61.4	110	53.4	reference		
CT	127	33.3	88	42.7	**1.47**	**1.03**–**2.08**	**0.032**
TT	20	5.2	8	3.9	0.85	0.36–2.00	0.710
T allele	147	—	96	—	1.39	0.99–1.96	0.060
C allele^*^	361	—	198	—	0.73	0.32–1.69	0.460
rs7745775 (T>G)							
TT	214	56.3	131	63.3	reference		
TG	145	38.2	69	33.3	0.78	0.54–1.11	0.170
GG	21	5.5	7	3.4	0.54	0.23–1.32	0.177
G allele	166	—	76	—	0.75	0.53–1.06	0.102
T allele^*^	359	—	200	—	0.60	0.25–1.43	0.249
**rs9364554** (C>T)							
CC	223	58.8	116	56.0	reference		
CT	144	38.0	77	37.2	1.03	0.72–1.47	0.879
TT	12	3.2	14	6.8	**2.22**	**1.01**–**5.00**	**0.049**
T allele	156	—	91	—	1.12	0.79–1.59	0.512
C allele^*^	367	—	193	—	**2.22**	**1.01**–**5.00**	**0.048**
**rs2457571** (T>C)							
TT	103	27.5	46	22.2	reference		
TC	209	55.9	111	53.6	1.19	0.78–1.82	0.415
CC	62	16.6	50	24.2	**1.82**	**1.09**–**3.03**	**0.023**
C allele	271	—	161	—	1.33	0.89–2.00	0.160
T allele^*^	312	—	157	—	**1.61**	**1.55**–**2.44**	**0.027**
rs7758229 (G>T)							
GG	184	48.7	104	50.5	reference		
GT	163	43.1	83	40.3	0.90	0.63–1.28	0.567
TT	31	8.2	19	9.2	1.09	0.58–2.00	0.798
T allele	194	—	102	—	0.93	0.66–1.30	0.676
G allele^*^	347	—	187	—	1.14	0.63–2.08	0.673
rs12527649 (G>A)							
GG	336	88.7	191	91.8	reference		
GA	41	10.8	16	7.7	0.68	0.37–1.25	0.223
AA	2	0.5	1	0.5	0.88	0.08–10.00	0.917
A allele	43	—	17	—	0.69	0.39–1.25	0.227
G allele^*^	377	—	207	—	0.91	0.08–10.00	0.939
rs4708867 (A>G)							
AA	307	81.6	164	78.8	reference		
AG	63	16.8	41	19.7	1.22	0.79–1.89	0.375
GG	6	1.6	3	1.4	0.93	0.23–3.85	0.926
G allele	69	—	44	—	1.19	0.78–1.82	0.412
A allele^*^	370	—	205	—	0.90	0.22–3.70	0.885
rs17593921 (C>T)							
CC	353	93.4	198	95.7	reference		
CT	25	6.6	9	4.3	0.64	0.29–1.41	0.266
TT	0	0	0	0	NA	NA	NA
T allele	25	—	9	—	0.64	0.29–1.41	0.266
C allele^*^	378	—	207	—	NA	NA	NA
rs1397168 (A>T)							
AA	263	69.2	142	68.3	reference		
AT	109	28.7	62	29.8	1.05	0.72–1.54	0.784
TT	8	2.1	4	1.9	0.93	0.27–3.13	0.902
T allele	117	—	66	—	1.04	0.72–1.52	0.814
A allele^*^	372	—	204	—	0.91	0.27–3.03	0.881
rs3088441 (C>T)							
CC	321	84.5	179	86.5	reference		
CT	58	15.3	28	13.5	0.86	0.53–1.41	0.561
TT	1	0.3	0	0	NA	NA	NA
T allele	1	—	0	—	0.85	0.52–1.39	0.515
C allele^*^	322	—	179	—	NA	NA	NA
rs2504926 (C>T)							
CC	107	28.4	63	30.6	reference		
CT	198	52.5	110	53.4	0.94	0.64–1.39	0.770
TT	72	19.1	33	16.0	0.78	0.47–1.30	0.342
T allele	270	—	143	—	0.90	0.62–1.30	0.576
C allele^*^	305	—	173	—	0.81	0.52–1.27	0.356

^*^Rare type genotype as reference.

N = numbers of individuals, OR = odds ratio, 95% CI = 95% confidence interval.

Missing genotypes are due to due to inadequate quantity or quality of DNA. Rs12212246 SNP was not analyzed due to technical reasons and rs3004079 due to its deviation from Hardy-Weinberg equilibrium as described in Patients and Methods.

Significant results and SNPs assessed in the validation phase are in bold.

**Table 3:** Results of validation study of putative loci in *SLC22A3* associating with pancreatic cancer risk in the discovery phase.

SNP	Controls		Cases		Crude analyses		p	Adjusted analyses^*^		p
genotype	N^#^	%	N^#^	%	OR	95% CI		aOR	95% CI	
rs2504956 (C>T)										
CC	1817	61.7	632	61.4	reference			reference		
CT	986	33.5	350	34.0	0.98	0.84–1.14	0.793	1.07	0.94–1.27	0.395
TT	141	4.8	48	4.7	1.02	0.73–1.43	0.901	1.17	0.81–1.69	0.398
T allele	1127	—	398	—	0.98	0.85–1.14	0.838	1.09	0.93–1.27	0.305
C allele^†^	2803	—	982	—	0.97	0.69–1.35	0.867	1.16	0.81–1.66	0.427
rs512077 (A>G)										
AA	2101	70.5	726	70.1	reference			reference		
AG	809	27.2	280	27.0	0.99	0.85–1.18	0.984	0.95	0.80–1.13	0.596
GG	69	2.3	30	2.9	0.79	0.51–1.23	0.303	1.07	0.66–1.72	0.784
G allele	878	—	310	—	0.98	0.84–1.14	0.785	0.96	0.82–1.14	0.660
A allele^†^	2910	—	1006	—	1.26	0.81–1.92	0.301	1.08	0.67–1.74	0.755
rs2504938 (C>T)										
CC	1733	59.0	631	60.6	reference			reference		
CT	1055	35.9	358	34.4	1.07	0.92–1.25	0.359	1.01	0.86–1.19	0.929
TT	149	5.1	52	5.0	1.04	0.75–1.45	0.800	1.15	0.81–1.62	0.437
T allele	1204	—	410	—	1.07	0.93–1.23	0.364	1.02	0.88–1.20	0.771
C allele^†^	2788	—	989	—	0.98	0.71–1.35	0.921	1.15	0.77–1.52	0.433
rs9364554 (C>T)										
CC	1792	62.5	597	60.2	reference			reference		
CT	912	31.8	340	34.3	0.89	0.77–1.04	0.156	**1.20**	**1.01**–**1.42**	**0.034**^**‡**^
TT	162	5.7	55	5.5	0.98	0.71–1.35	0.908	1.15	0.81–1.65	0.429
T allele	1074	—	395	—	0.91	0.78–1.05	0.190	**1.19**	**1.02**–**1.40**	**0.030**^**‡**^
C allele^†^	2704	—	937	—	0.98	0.71–1.33	0.899	1.08	0.77–1.52	0.647
rs2457571 (T>C)										
TT	885	30.8	327	32.1	reference			reference		
TC	1431	49.8	485	47.5	1.09	0.93–1.28	0.300	1.04	0.87–1.24	0.645
CC	559	19.4	208	20.4	0.99	0.81–1.20	0.946	1.21	0.97–1.51	0.097
C allele	1990	—	693	—	1.06	0.91–1.23	0.449	1.09	0.93–1.29	0.296
T allele^†^	2316	—	812	—	1.06	0.89–1.27	0.513	1.18	0.97–1.44	0.093
rs7758229 (G>T)										
GG	1637	55.3	568	54.7	reference			reference		
GT	1121	37.8	403	38.8	0.96	0.83–1.12	0.640	1.13	0.96–1.33	0.138
TT	204	6.9	68	6.5	1.04	0.78–1.41	0.787	1.00	0.73–1.39	0.981
T allele	1325	—	471	—	0.98	0.85–1.14	0.738	1.11	0.95–1.30	0.174
G allele^†^	2962	—	971	—	0.95	0.71–1.27	0.706	0.97	0.71–1.32	0.826

^*^Adjusted to age, sex, and country of origin.

^#^N = number of individuals.

^†^Rare genotype as reference.

^‡^Result did not pass the FDR test for multiple comparisons (q = 0.008).

Significant results are in bold.

**Table 4:** Analyses of distribution of *SLC22A3* SNPs in pancreatic cancer patients stratified by the presence of distant metastases in the discovery phase.

SNP	M0		M1		OR	95% CI	p
genotype	N	%	N	%			
rs7758229							
GG	33	66.0	19	41.3	reference		
**GT**	**11**	**22.0**	**23**	**50.0**	**3.63**	**1.46**–**9.06**	**0.006**
TT	6	12.0	4	8.7	1.16	0.29–4.63	0.836
**T alelle**	**17**	—	**27**	—	**2.76**	**1.20**–**6.32**	**0.016**
G allele^*^	44	—	42	—	0.70	0.18–2.65	0.598

^*^TT genotype as reference.

N = numbers of individuals, OR = odds ratio, 95% CI = 95% confidence interval.

Missing genotypes are due to due to inadequate quantity or quality of DNA or due to missing data. All SNPs were analyzed but to retain concise style only significant associations are reported. Significant results are in bold.
