# Supplementary material for: SLC22A3 polymorphisms do not modify pancreatic cancer risk, but may influence overall patient survival
Source: Sci Rep. 2017 Mar 8;7:43812. doi: 10.1038/srep43812 (PMC5341046; doi:10.1038/srep43812)

***SLC22A3* polymorphisms do not modify pancreatic cancer risk, but may influence overall patient survival**

Beatrice Mohelnikova-Duchonova^1,2*^, Ondrej Strouhal^2^, David J. Hughes^3^, Ivana Holcatova^4^, Martin Oliverius^5^, Zdenek Kala^6^, Daniele Campa^7,8^, Cosmeri Rizzato^7,9^, Federico Canzian^7^, Raffaele Pezzilli^10^, Renata Talar-Wojnarowska^11^, Ewa Malecka-Panas^11^, Cosimo Sperti^12^, Carlo Federico Zambon^13^, Sergio Pedrazzoli^14^, Paola Fogar^15^, Anna Caterina Milanetto^12^, Gabriele Capurso^16^, Gianfranco Delle Fave^16^, Roberto Valente^16^, Maria Gazouli^17^, Giuseppe Malleo^18^, Rita Teresa Lawlor^19^, Oliver Strobel^20^, Thilo Hackert^20^, Nathalia Giese^20^, Pavel Vodicka^21,25^, Ludmila Vodickova^21,25^, Stefano Landi^8^, Francesca Tavano^22^, Domenica Gioffreda^22^, Ada Piepoli^22^, Valerio Pazienza^22^, Andrea Mambrini^23^, Mariangela Pedata^23^, Maurizio Cantore^23^, Franco Bambi^24^, Stefano Ermini^24^, Niccola Funel^9^, Pavel Soucek^1,25^.

^1^Department of Toxicogenomics, National Institute of Public Health, Prague, Czech Republic

^2^Department of Oncology, Palacky University Medical School and Teaching Hospital, Olomouc, Czech Republic

^3^Department of Physiology & Centre for Systems Medicine, Royal College of Surgeons in Ireland, Dublin 2, Ireland

^4^Institute of Hygiene and Epidemiology, First Faculty of Medicine, Charles University in Prague, Prague, Czech Republic

^5^Department of Transplantation Surgery, Institute of Clinical and Experimental Medicine, Prague, Czech Republic

^6^Department of Surgery, The University Hospital and Faculty of Medicine, Brno Bohunice, Czech Republic

^7^Genomic Epidemiology Group, German Cancer Research Center (DKFZ), Heidelberg, Germany

^8^Department of Biology, University of Pisa, Pisa, Italy

^9^Department of Translational Research and New Technologies in Medicine and Surgery, University of Pisa, Pisa, Italy

**^10^Department of Digestive Diseases, Sant'Orsola-Malpighi Hospital, Bologna, Italy**

**^11^Department of Digestive Tract Diseases, Medical University of Lodz, Lodz, Poland**

^12^Department of Surgery, Oncology and Gastroenterology -DiSCOG, University of Padova, Italy
^13^Department of Medicine - DIMED, University of Padova, Italy
^14^Clinica Chirurgica 4, University of Padova, Italy
^15^Department of Laboratory Medicine, University-Hospital of Padova, Italy

^16^Digestive and Liver Disease Unit, S. Andrea Hospital, ‘Sapienza’ University of Rome, Rome, Italy

^17^Department of Basic Medical Sciences, Laboratory of Biology, School of Medicine, University of Athens, Athens, Greece

^18^Department of Surgery and Oncology, University and Hospital Trust of Verona, Verona, Italy

^19^ARC-NET Applied research on Cancer Centre, University and Hospital Trust of Verona, Verona, Italy

^20^Department of General, Visceral and Transplantation Surgery, Heidelberg University Hospital, Heidelberg, Germany

^21^Department of Molecular Biology of Cancer, Institute of Experimental Medicine, Academy of Science of Czech Republic, Prague, Czech Republic and First Faculty of Medicine, Charles University in Prague, Czech Republic

^22^Division of Gastroenterology and Research Laboratory, IRCCS Scientific Institute and Regional General Hospital “Casa Sollievo della Sofferenza”, San Giovanni Rotondo, Italy

^23^Department of Oncology, Azienda USL 1 Massa Carrara, Massa Carrara, Italy

^24^Blood Transfusion Service, Children's Hospital Meyer, Azienda Ospedaliero Universitaria, Florence, Italy

**^25^**Biomedical Centre, Faculty of Medicine in Pilsen, Charles University in Prague, Pilsen, Czech Republic

**Running title:** Role of SLC22A3 polymorphisms in pancreatic cancer.

**Keywords**: pancreas; cancer risk; polymorphisms; SLC22A3; survival

***Corresponding author**: Beatrice Mohelnikova-Duchonova, MD, PhD, Department of Oncology, Palacky University Medical School and Teaching Hospital, Olomouc, Czech Republic, email: [d.beatrice@seznam.cz](mailto:d.beatrice@seznam.cz)

**Supplementary Table S1:** Results of combined analysis of putative loci in *SLC22A3*

SNP

Genotype

Controls

Cases

Crude analyses

N

N

OR

Lower 95% CI

Upper 95 % CI

p

**rs2504956**

CC

2 053

745

reference

CT

1 105

430

0.93

0.81

1.07

0.326

TT

158

58

0.99

0.72

1.35

0.942

T allele

1 263

488

0.94

0.82

1.07

0.359

C allele

3 158

1 175

0.99

0.72

1.34

0.932

**rs512077**

AA

2 349

874

reference

AG

916

333

1.02

0.88

1.19

0.758

GG

79

35

0.84

0.56

1.26

0.399

G allele

995

368

1.01

0.87

1.16

0.934

A allele

3 265

1 207

1.20

0.80

1.79

0.181

**rs2504938**

CC

1 962

740

reference

CT

1 180

445

1.00

0.87

1.15

0.999

TT

168

60

1.06

0.78

1.44

0.727

T allele

1 348

505

1.01

0.88

1.15

0.921

C allele

3 142

1 185

0.95

0.70

1.28

0.724

**rs9364554**

CC

2 009

713

reference

CT

1 052

416

0.90

0.78

1.03

0.136

TT

174

69

0.89

0.67

1.20

0.456

T allele

1 226

485

0.90

0.78

1.03

0.116

C allele

3 061

1 129

1.08

0.81

1.43

0.621

**rs2457571**

TT

985

373

reference

TC

1 634

594

1.04

0.90

1.21

0.598

CC

621

258

0.91

0.76

1.10

0.333

C allele

2 255

852

1.00

0.87

1.16

0.975

T allele

2 619

967

1.13

0.96

1.32

0.156

**rs7758229**

GG

1 814

671

reference

GT

1 282

485

0.98

0.85

1.12

0.748

TT

231

87

0.98

0.76

1.28

0.893

T allele

1 513

572

0.98

0.86

1.12

0.744

G allele

3 096

1 156

1.01

0.78

1.30

0.947

**Supplementary Table S2:** Results of adjusted analyses of associations of *SLC22A3* SNPs with pancreatic cancer risk adjusted for major lifestyle factors in the discovery phase

SNP aOR 95% CI p

genotype

rs316174 (A>G)

AA reference

AG 1.22 0.70-2.13 0.480

GG 0.75 0.36-1.55 0.436

G allele 1.08 0.63-1.84 0.783

A allele^*^ 0.79 0.43-1.44 0.440

rs2504956 (C>T)

CC reference

CT 1.27 0.76-2.10 0.360

TT 2.09 0.65-6.69 0.214

T allele 1.33 0.82-2.16 0.243

C allele^*^ 1.89 0.62-5.78 0.265

rs572149 (A>G)

AA reference

AG 0.85 0.52-1.40 0.518

GG 0.74 0.30-1.78 0.494

G allele 0.83 0.51-1.34 0.439

A allele^*^ 0.83 0.36-1.90 0.657

rs3120137 (G>A)

GG reference

GA 0.78 0.44-1.36 0.376

AA 1.27 0.16-10.33 0.823

A allele 0.80 0.46-1.39 0.424

G allele^*^ 1.35 0.17-10.82 0.780

rs512077 (A>G)

AA reference

AG 1.07 0.62-1.84 0.816

GG 0.82 0.18-3.87 0.821

G allele 1.05 0.62-1.77 0.857

A allele^*^ 0.83 0.18-3.80 0.807

rs675162 (A>G)

AA reference

AG 1.38 0.77-2.48 0.284

GG 0.93 0.45-1.91 0.835

G allele 1.27 0.72-2.23 0.406

A allele^*^ 0.77 0.43-1.36 0.359

rs394487 (C>T)

CC reference

CT 0.75 0.46-1.23 0.259

TT 0.56 0.20-1.30 0.271

T allele 0.72 0.45-1.57 0.173

C allele^*^ 0.65 0.24-1.76 0.400

**Supplementary Table S2:** Results of adjusted analyses of associations of *SLC22A3* SNPs with pancreatic cancer risk adjusted for major lifestyle factors in the discovery phase – continued

SNP aOR 95% CI p

genotype

rs10455871(A>T)

AA reference

AT 1.68 0.30-9.42 0.558

TT NA NA NA

T allele 1.68 0.30-9.42 0.558

A allele^*^ NA NA NA

rs884742 (C>A)

CC reference

CA 1.49 0.83-2.65 0.180

AA 0.97 0.47-2.02 0.935

A allele 1.37 0.78-2.39 0.276

C allele^*^ 0.72 0.40-1.30 0.276

rs420038 (C>T)

CC reference

CT 0.71 0.43-1.17 0.181

TT 0.85 0.31-2.32 0.744

T allele 0.72 0.44-1.17 0.576

C allele^*^ 1.00 0.38-2.61 0.998

rs1567441 (T>C)

TT reference

TC 0.72 0.44-1.18 0.188

CC 0.94 0.31-2.83 0.909

C allele 0.74 0.46-1.20 0.216

T allele^*^ 1.11 0.39-3.21 0.842

**rs2504938** (C>T)

CC reference

**CT 1.72 1.06-2.81 0.030**

TT 0.78 0.21-2.80 0.688

**T allele** **1.61 1.00-2.57 0.049**

C allele^*^ 0.68 0.20-2.34 0.540

rs7745775 (T>G)

TT reference

TG 0.85 0.52-1.40 0.518

GG 0.51 0.15-1.68 0.268

G allele 0.82 0.51-1.33 0.420

T allele^*^ 0.54 0.16-1.74 0.299

rs9364554 (C>T)

CC reference

CT 1.00 0.62-1.63 0.971

TT 2.20 0.67-7.15 0.192

T allele 1.08 0.68-1.73 0.742

C allele^*^ 2.34 0.72-7.63 0.160

**Supplementary Table S2:** Results of adjusted analyses of associations of *SLC22A3* SNPs with pancreatic cancer risk adjusted for major lifestyle factors in the discovery phase – continued

SNP aOR 95% CI p

genotype

**rs2457571** (T>C)

TT reference

TC 0.88 0.49-1.58 0.677

CC 1.85 0.91-3.74 0.089

C allele 1.11 0.64-1.93 0.711

**T allele^*^ 1.93 1.10-3.40 0.022**

rs7758229 (G>T)

GG reference

GT 0.86 0.52-1.41 0.542

TT 1.03 0.42-2.51 0.974

T allele 0.89 0.55-1.43 0.632

G allele^*^ 1.10 0.47-2.56 0.825

rs12527649 (G>A)

GG reference

GA 0.52 0.23-1.21 0.130

AA 0.52 0.04-6.76 0.614

A allele 0.52 0.23-1.16 0.112

G allele^*^ 0.52 0.04-6.66 0.619

**rs4708867** (A>G)

AA reference

AG 2.02 1.09-3.72 0.025

GG 0.80 0.88-7.29 0.844

**G allele 1.89 1.05-3.42 0.035**

A allele^*^ 0.68 0.08-6.19 0.732

rs17593921 (C>T)

CC reference

CT 0.88 0.28-2.74 0.826

TT NA NA NA

T allele 0.88 0.28-2.74 0.826

C allele^*^ NA NA NA

rs1397168 (A>T)

AA reference

AT 1.52 0.91-2.56 0.112

TT 0.50 0.10-2.51 0.399

T allele 1.41 0.85-2.32 0.181

A allele^*^ 0.48 0.09-2.40 0.367

rs3088441 (C>T)

CC reference

CT 0.75 0.37-1.53 0.428

TT NA NA NA

T allele 0.75 0.37-1.53 0.428

C allele^*^ NA NA NA

**Supplementary Table S2:** Results of adjusted analyses of associations of *SLC22A3* SNPs with pancreatic cancer risk adjusted for major lifestyle factors in the discovery phase – continued

SNP aOR 95% CI p

genotype

rs2504926 (C>T)

CC reference

CT 0.75 0.44-1.28 0.291

TT 0.59 0.28-1.25 0.170

T allele 0.72 0.43-1.19 0.197

C allele^*^ 0.75 0.40-1.42 0.378

Footnotes:

^*^Rare type genotype as reference

N=numbers of individuals, aOR = adjusted odds ratio, 95% CI = 95% confidence interval. Analyses were adjusted for age, sex, body mass index, smoking status, and alcohol consumption.

Missing genotypes are due to due to inadequate quantity or quality of DNA. Rs12212246 SNP was not analyzed due to technical reasons and rs3004079 due to its deviation from Hardy- Hardy-Weinberg equilibrium as described in Patients and Methods.

Significant results and SNPs assessed in the validation phase are in bold.

**Supplementary Table S3:** Stage-adjusted Cox regression analysis of associations between SNPs in *SLC22A3* and OS of pancreatic cancer patients (only significant associations in univariate analyses tested)

**Discovery set (Figures 1 and 2)**

SNP HR* 95% CI* p-value

rs512077

AA genotype reference

G allele 1.08 0.72 – 1.61 0.710

rs2504956

CC genotype reference

T allele 0.81 0.56 – 1.16 0.247

rs2504938

CC genotype reference

TT genotype 0.49 0.17 – 1.33 0.163

**Validation set (Figure 2)**

SNP HR* 95% CI* p-value

rs512077

AA genotype reference

G allele 0.94 0.75-1.19 0.630

rs2504956

CC genotype reference

T allele 0.91 0.75-1.14 0.426

rs2504938

CC genotype reference

TT genotype 1.37 0.84-2.22 0.205

*HR = hazard ratio, 95% CI = 95% confidence interval

**Supplementary Table S4:** *SLC22A3* SNPs analyzed in the study

| Footnotes: |
| --- |
| ^*^Checked by Mutalyzer v2.0 (https://mutalyzer.nl/snp) according to Wildeman et al. Improving sequence variant descriptions in mutation databases and literature using the Mutalyzer sequence variation |
| ^#^MAF = minor allele frequency in EUR sub-populations (CEU) according to 1000 Genomes data (<http://www.1000genomes.org/>)  SNP=single nucleotide polymorphism; HGVS=human genome variation society |

**Supplementary Table S5:** SLC22A3 SNP assays and conditions

SNP

Assay number*

Assay conditions

rs2504956

C_16252836_10

10 min @ 95

o

C//40 cycles consisting of 15 sec @ 95

o

C/60 sec @60

o

C

rs512077

C_717602_10

10 min @ 95

o

C//40 cycles consisting of 15 sec @ 95

o

C/60 sec @60

o

C

rs2457571

C_2737075_10

10 min @ 95

o

C//40 cycles consisting of 15 sec @ 95

o

C/60 sec @60

o

C

rs7758229

C_2737078_10

10 min @ 95

o

C//40 cycles consisting of 15 sec @ 95

o

C/60 sec @60

o

C

rs9364554

C_2737073_10

10 min @ 95

o

C//40 cycles consisting of 15 sec @ 95

o

C/60 sec @60

o

C

rs2504938

C_16252817_20

10 min @ 95

o

C//40 cycles consisting of 15 sec @ 95

o

C/60 sec @60

o

C

*Assay purchased from Life Technologies, Foster City, CA, USA

**Supplementary Figure S1**: Association of *SLC22A3* rs2504938 SNP with overall survival of PDAC patients in both phases combined

Kaplan–Meier survival curves for patients with CC genotype (solid line) *vs.* patients with TT genotype (dashed line) are displayed. The difference in the mean survival between the compared groups of patients was not significant (p=0.073). Hazard ratios, 95% confidence intervals, and p-values calculated by the stage-adjusted Cox regression are presented in **Supplementary Table S3**.


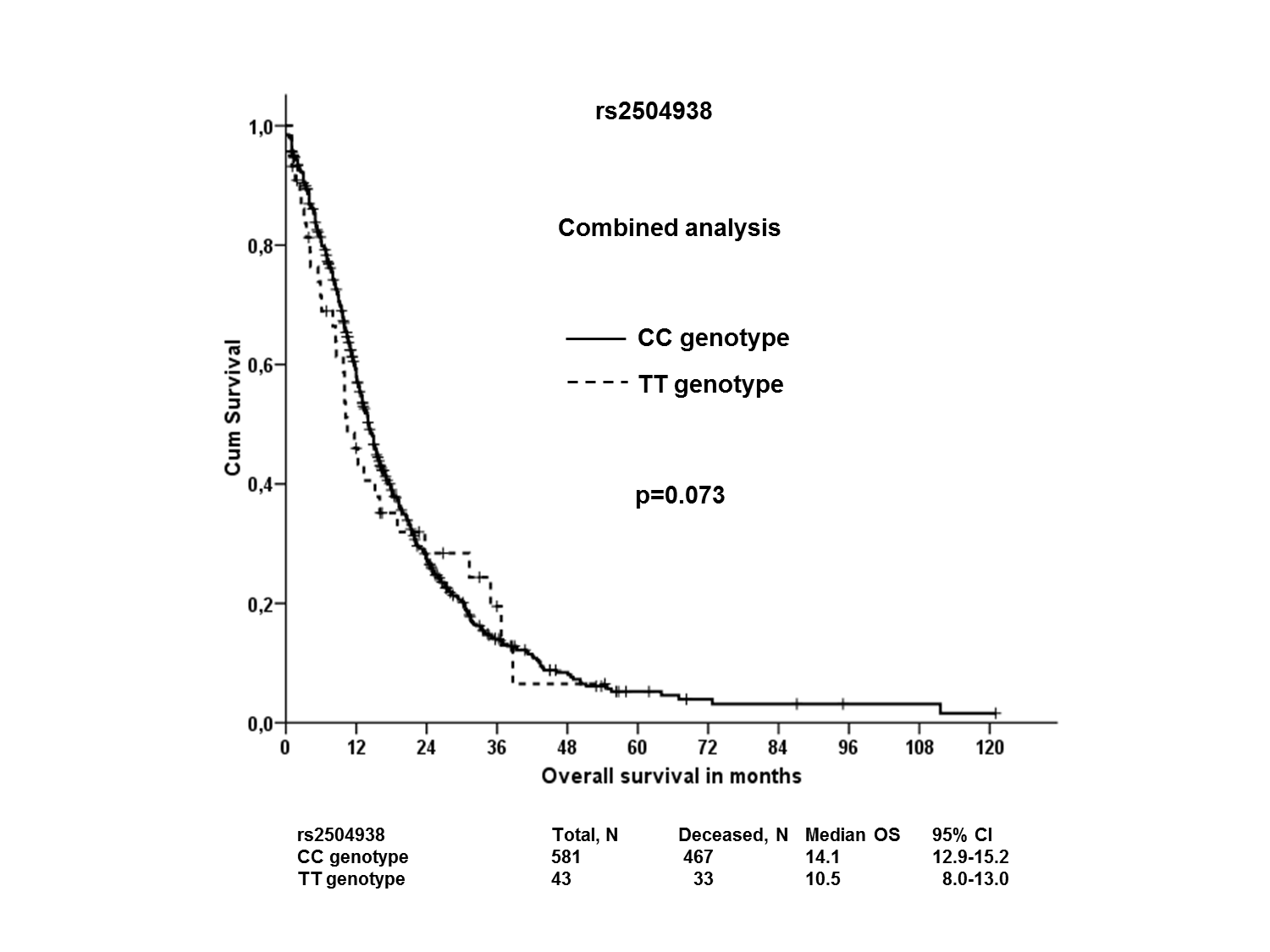

Supplement: Supplementary Dataset 1 [file srep43812-s1.doc]
